# Supplementary material for: Large Questionnaire Survey on Sleep Duration and Insomnia Using the TV Hybridcast System by Japan Broadcasting Corporation (NHK)
Source: Int J Environ Res Public Health. 2021 Mar 7;18(5):2691. doi: 10.3390/ijerph18052691 (PMC7967419; doi:10.3390/ijerph18052691)
Supplement: Supplementary file 1 [file ijerph-18-02691-s001.pdf]

Supplementary table S1. Comparison of recording time of responses and sleep parameters

	During broadcast	Not at the time of broadcast	Cramer's V
Sleep duration (hours)			
< 5	18403 (12.3%)	11532 (7.6%)	0.099
5-6	44249 (29.6%)	40317 (26.5%)	
6-7	55626 (37.2%)	67889 (44.7%)	
7-8	22875 (15.3%)	24092 (15.9%)	
8-9	6501 (4.4%)	6271 (4.1%)	
≥ 9	1706 (1.2%)	1780 (1.2%)	
AIS			
0-5	64707 (43.3%)	57318 (37.7%)	0.067
6-9	56574 (37.9%)	59105 (38.9%)	
10-15	25026 (16.8%)	31593 (20.8%)	
16-24	3053 (2.0%)	3865 (2.5%)	

Cramer's V was calculated to present effect size of association between time of recording responses and sleep duration and AIS score categories. Cramer's V of 0.1 or larger suggests small effect.

AIS: Athens insomnia scale
